# Supplementary material for: Halanaerobium polyolivorans sp. nov.—A Novel Halophilic Alkalitolerant Bacterium Capable of Polyol Degradation: Physiological Properties and Genomic Insights
Source: Microorganisms. 2023 Sep 15;11(9):2325. doi: 10.3390/microorganisms11092325 (PMC10536098; doi:10.3390/microorganisms11092325)
Supplement: Supplementary file 1 [file microorganisms-11-02325-s001.zip › microorganisms-2534316-supplementary.pdf]

Halanaerobium polyolivorans sp. nov.—A Novel Halophilic Alkalitolerant Bacterium Capable of Polyol Degradation: Physiological Properties and Genomic Insights

Yulia Boltyanskaya, Tatjana Zhilina, Denis Grouzdev, Ekaterina Detkova, Nikolay Pimenov and Vadim Kevbrin

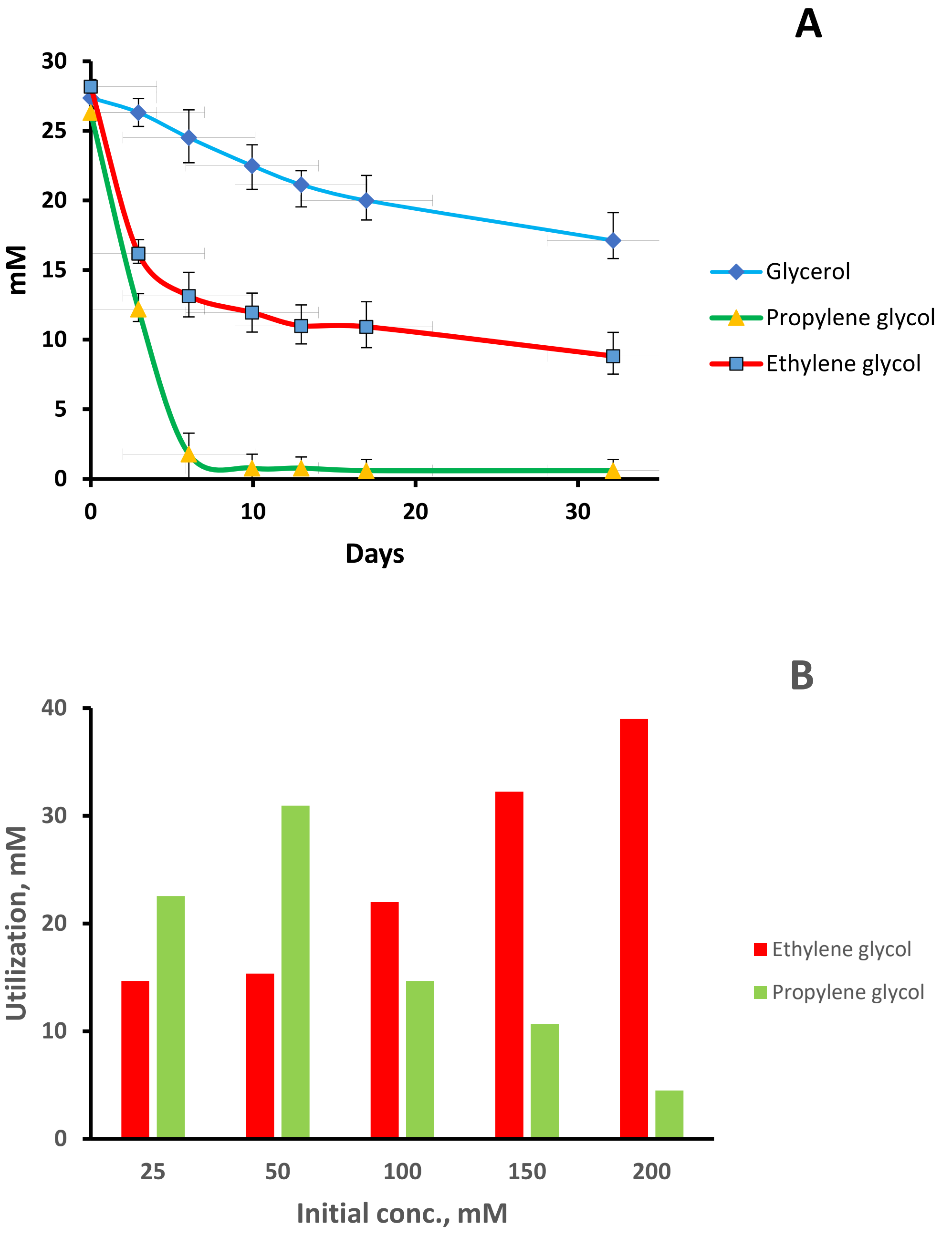

Figure S1. (**A**) Polyol utilization ($C_{residual}$) by strain Z-7514[T] in course of time at a given initial concentration of 25 mM each; (**B**) polyol utilization ($C_{ini} - C_{fin}$) at given initial concentrations.
